# Use of carbon dioxide production to detect bacterial superinfections in mechanically ventilated patients with acute respiratory distress syndrome: an exploratory prospective cohort study

**DOI:** 10.1136/bmjresp-2024-002760

**Published:** 2025-08-28

**Authors:** Nicole S Strickler, Daniel A Hofmaenner, Reto A Schuepbach, Thomas C Scheier, Philipp K Buehler, Jukka Takala, Silvio D Brugger, Pascal M Frey

**Affiliations:** 1 Department of Infectious Diseases and Hospital Epidemiology, University Hospital Zurich, Zurich, Switzerland; 2 Institute of Intensive Care, University Hospital Zurich, Zurich, Switzerland; 3 Institute of Intensive Care, Kantonsspital Winterthur, Winterthur, Switzerland; 4 University of Bern, Bern, Switzerland; 5 Department of General Internal Medicine, Inselspital University Hospital Bern, Bern, Switzerland

**Keywords:** ARDS, Bacterial Infection, COVID-19, Critical Care, Pneumonia, Respiratory Infection

## Abstract

**Background:**

Bacterial superinfections are common in patients with acute respiratory distress syndrome (ARDS) but diagnosing them is challenging. Exhaled carbon dioxide (V’CO2) may be increased during bacterial infection, suggesting a potential marker for detecting bacterial superinfections in ARDS patients.

**Methods:**

In a prospective cohort study of mechanically ventilated adult patients with ARDS due to SARS-CoV-2 in a tertiary intensive care unit, we assessed V’CO2 measurements from continuous volumetric capnography and calculated daily median V’CO2 levels. The primary outcome was to determine if a first substantial increase in daily median V’CO2 was associated with a first bacterial superinfection. Protocolised microbiological sampling and adjudicated clinical interpretations were used to determine the onset of a first superinfection.

**Results:**

A total of 150 days of continuous volumetric capnography were analysed in 31 mechanically ventilated adult patients with ARDS due to SARS-CoV-2. We observed 10 patients (32%) with a first episode of substantial increase of daily median V’CO2, and 12 (39%) patients with a first bacterial superinfection. A V’CO2 increase was not associated with a superinfection on the same day (OR 3.47, 95% CI 0.64 to 18.92, p=0.15, adjusted for age and gender). Investigating all 150 test days of median V'CO2 revealed a poor sensitivity (17%, 95% CI 2% to 48%) for detecting superinfections. However, a first V'CO2 increase indicated superinfection with high specificity (94%, 95% CI 89% to 98%). Patients with superinfections showed higher daily median V'CO2 levels (210 mL/min) than those without (176 mL/min, p<0.001), even after adjusting for age and gender (OR 1.56, 95% CI 1.16 to 2.08, p=0.003).

**Conclusions:**

A sudden increase in daily median V’CO2 did not reliably detect bacterial superinfections, which was reflected in a poor sensitivity and inability to rule out superinfections in patients without V’CO2 increase. Nevertheless, high specificity suggests that V’CO2 may be useful to rule in superinfections in patients with ARDS.

**Trial registration number:**

NCT04410263.

**WHAT IS ALREADY KNOWN ON THIS TOPIC**

- Detecting bacterial superinfection in patients with acute respiratory distress syndrome (ARDS) is often challenging but essential to initiate appropriate treatment in a timely manner, underscoring the necessity of additional markers to aid clinicians. Exhaled carbon dioxide (V’CO2) from continuous capnography is known to increase in some bacterial infections, therefore, we aimed to investigate its utility to detect superinfections in patients with ARDS.

**WHAT THIS STUDY ADDS**

- A rapid increase of at least 20% in V’CO2 is likely to be of limited use for the detection of superinfections, as the lack of an increase does not rule out superinfections due to low sensitivity. Nevertheless, specificity was high, making it a potentially useful marker to rule in the presence of a superinfection in ARDS.

**HOW THIS STUDY MIGHT AFFECT RESEARCH, PRACTICE OR POLICY**

- While clinicians should be aware of bacterial superinfections in the presence of a sudden V’CO2 increase, further research may focus on using V’CO2 as a rule-in criteria in conjunction with clinical and laboratory markers to improve the timely diagnosis of superinfections in patients with ARDS.

## Introduction

 Clinically relevant secondary bacterial infections (superinfections) are frequent and associated with longer ventilation times, increased duration of intensive care and hospitalisation in mechanically ventilated acute respiratory distress syndrome (ARDS) patients.^1^,^3^ High rates of pulmonary superinfections have also been described recently in patients suffering from ARDS due to COVID-19.^4^,^7^ An uncertain prevalence of bacterial superinfections in ARDS patients and the occasional detection of Gram-negative bacteria including multidrug resistant pathogens might lead to increased use of reserve antibiotics. Therefore, an early and reliable detection of bacterial superinfections and implementation of antibiotic stewardship is critical not only to lower morbidity and mortality in ARDS patients in general, but also to reduce the selection of antimicrobial resistance. To date, no validated biomarker to predict a clinically relevant bacterial superinfection in ARDS patients has, to our knowledge, been identified.

Exhaled carbon dioxide output (V’CO_2_) is defined as the amount of V’CO_2_ from the body per unit time and is expressed in ml/min. V’CO_2_ is provided by modern ventilators with volumetric capnography and is calculated by VeCO_2_ (volume of CO_2_ exhaled at each breath) multiplied by the respiratory rate.^8^ V’CO_2_ equals the CO_2_ production in steady, stable conditions of circulation and ventilation.^9^ Often, V’CO2 from volumetric capnography has been used to investigate energy expenditure,^10^,^12^ in comparison to V’CO2 results using indirect calorimetry,^13 14^ whereas indirect calorimetry is still considered the gold standard to define energy expenditure. An increase in V’CO_2_ can be observed in hypermetabolic states such as sepsis.^15^,^17^ Similarly, a general hypermetabolism with increased V’CO_2_ has been observed in COVID-19 patients,^18^ with sometimes also an increase of both V’CO2 and oxygen consumption (V’O2).^19^ Two studies examined that changes in carbon dioxide production measured by indirect calorimetry have been observed in patients with sepsis.^17 20^ Other studies have analysed V’CO2 in similar contexts such as metabolic changes during septic shock.^16 21^ Bacterial superinfections could be assumed to induce or worsen hypermetabolism, as has been shown in sepsis,^15^ and thus raise the question of whether metabolic surrogates such as V’CO2 levels could potentially be used to predict bacterial superinfections in critically ill COVID-19 patients. On the other hand, superinfections could also increase the lung metabolic activity, thus increasing V’O2.^22^

However, to our knowledge, there have been no studies investigating if V’CO2 routinely measured during mechanical ventilation was associated with bacterial superinfections in ARDS patients. Thus, our aim was to determine the association between a first episode of substantial increase (>20%) in daily median V’CO2 and a bacterial superinfection. We hypothesised that a substantial increase in daily median V’CO2 was associated with bacterial superinfection in mechanically ventilated adult ARDS patients.

## Methods

### Study design and population

To analyse a homogeneous group of similar ARDS patients, a recent cohort of critically ill COVID-19 patients was used to evaluate the study hypothesis. Patients were included from the MicrobiotaCOVID cohort study (ClinicalTrials.gov, NCT04410263, registered 1 June 2020), a single-centre, prospective cohort study to assess ARDS, bacterial superinfections and other outcomes in COVID-19 patients at the Institute of Intensive Care Medicine of the University Hospital Zurich, Switzerland.^4^

Patients were eligible if hospitalised with confirmed ARDS due to SARS-CoV-2 infection in the intensive care unit (ICU) between 1 January 2020 and 31 December 2020 during the first and second COVID-19 wave with invasive mechanical ventilation using a device allowing continuous V’CO2 measurements.

Inclusion criteria for our study were age ≥18 years, SARS-CoV-2 infection as determined by real-time reverse transcriptase-PCR (RT-PCR) positivity of nasopharyngeal and/or oropharyngeal swabs, tracheobronchial secretion (TBS) or bronchoalveolar lavage (BAL) and hospitalisation in the ICU for moderate or severe ARDS according to the Berlin criteria.^23^

Exclusion criteria were patients or relatives denying informed consent, patients still being treated in the ICU when the study period ended, invasive mechanical ventilation of less than 48 hours, extracorporeal life support (ECLS) and haemodialysis/filtration at any time of the ICU treatment.

### Data collection

As described previously,^4^ microbiological samples were collected by the ICU healthcare workers on ICU admission and during the later course of the disease. If the respiratory status permitted, as assessed by the attending ICU physician, BAL was performed by ICU staff on ICU admission and during the course of ICU stay using 10 mL of saline. TBS were collected from all ventilated patients at a minimum on day 0 (ICU admission), day 1, day 2, day 3, day 5 and subsequently every 5 days. Sampling was omitted if the clinical condition did not allow it.

Samples were processed at the Institute for Medical Microbiology and at the Institute for Medical Virology of the University of Zurich. Standard clinical microbiology techniques were used for culturing, isolation and identification of bacterial and fungal microorganisms as previously described.^24^ SARS-CoV-2 was detected by real-time RT-PCR.^25^

Clinical and laboratory data were obtained from electronic health records. V’CO2 was continuously measured (minimal interval 1 min) and obtained from electronic ventilator readouts (Hamilton ventilators S1 and C6, Hamilton Medical, Bonaduz, Switzerland). Baseline data included demographics, comorbidities, ICU and other scores (Sequential Organ Failure Assessment, Simplified Acute Physiology Score II, Charlson Comorbidity Index) and COVID-19 targeted experimental therapy (dexamethasone, remdesivir and plasma therapy) at baseline.

### Assessment of V’CO2 increase

Ventilator readouts with V’CO2 measurements every minute were aggregated to daily median V’CO2 measurements if there was an observational period of at least 1 hour (ie, >59 measurements per day), with a possible maximum of 1440 measurements per day. A daily median V’CO2 increase was considered substantial if the daily median was >20% higher than the daily median of the previous day. To our knowledge, no precise cut-off for daily median V’CO2 has been validated, thus the >20% increase was determined prior to data analysis by what a panel of ICU physicians regarded as clinically relevant. This also included considerations of the practicality of its recognition in the clinical environment by ICU physicians or nurses, ensuring it was substantial enough to be noticed in patient care situations.

### Study outcome

The primary study outcome was the association between a first episode of substantial increase in daily median V’CO2 and a first bacterial superinfection diagnosed on that same day. In a secondary post hoc analysis, we additionally investigated the association between absolute measures of V’CO2 and the following occurrence of a superinfection. A multidisciplinary panel of ICU and infectious diseases consultants assessed the clinical status of the patients on a daily basis. The diagnosis of superinfection was adjudicated by the panel based on clinical judgement and routine laboratory assessment as well as microbiological results as previously described.^4^ Although pulmonary superinfections were the predominant type observed, any new bacterial infection, regardless of organ system, was considered a potential superinfection and evaluated accordingly by the panel.

### Statistical analyses

Comparisons of population characteristics between patients with or without a clinically relevant V’CO2 increase were performed using Mann-Whitney U tests for continuous variables, or χ^2^/Fisher’s exact tests for categorical variables, as appropriate. To ascertain the association of V’CO2 and developing a superinfection, as well as a first clinically relevant V’CO2 increase and a first superinfection, we used logistic regression with adjustment for age and gender despite the low number of events.^26^

For clinical application, the sensitivity, specificity, as well as positive (LR+) and negative likelihood ratios (LR–) for a >20% increase in daily median V’CO2 and a superinfection on the same day were calculated. For the purpose of sensitivity and specificity calculations in our study, we considered a daily median V’CO2 measurement as a test, that was either positive (ie, have a ≥20% increase) or negative (ie, have no such increase), and could either correctly identify the presence of a first superinfection or not. Therefore, every day on which a daily median V’CO2 was measured (every day at risk of a first event) and a patient was at risk of experiencing either a first V’CO2 increase or a first superinfection was considered as a day the V’CO2 test was performed.

No formal sample size calculation was possible due to the novelty of our hypothesis and the absence of prior data on effect size for V’CO₂ dynamics. The study was based on a convenience sample from a prospective cohort and was considered exploratory in nature.

We considered a p value of <0.05 to be statistically significant, without adjustment for multiple hypothesis testing. All statistical analyses were performed using Stata 16 (Stata Corporation, College Station, TX, USA).

### Patient and public involvement

Patients and the public were not involved in the design, conduct, reporting or dissemination plans of this research.

## Results

### Cohort characteristics

**Figure 1:**
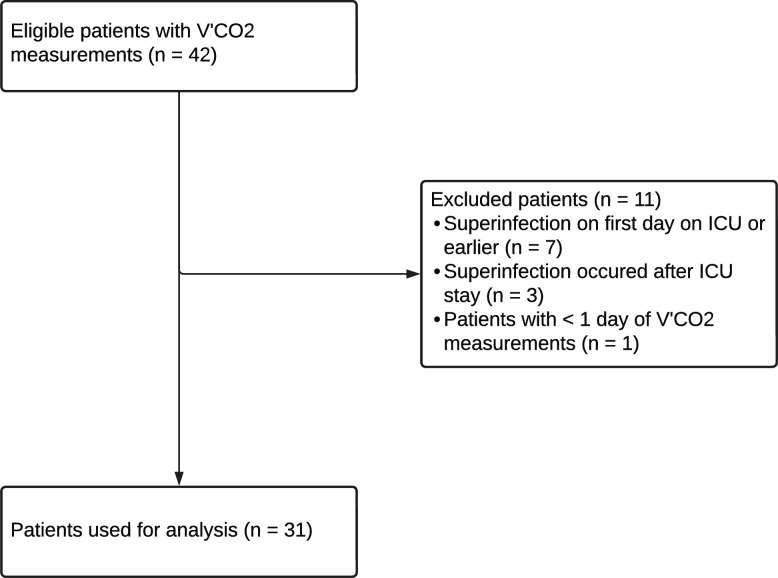
Study flow diagram. Flow diagram of exclusion process and in total analysed patients. ICU, intensive care unit; V’CO2, exhaled carbon dioxide.

Overall, 189 critically ill patients with confirmed ARDS initially included in the MicrobiotaCOVID cohort were discharged from the ICU at the University Hospital Zurich between January 2020 and December 2020, of which 42 patients had V’CO2 measurements recorded and were thus eligible for inclusion (no ECLS, no haemodialysis, informed consent). Of these, 11 patients were excluded from analysis either because the superinfection occurred on the first day of mechanical ventilation, the superinfection occurred after the ICU stay, or there were <2 days of V’CO2 measurements, therefore, inherently preventing the observation of a V’CO2 increase from 1 day to the next (figure 1).

Thus, 31 patients were included in the analysis, contributing to an overall test sample size of 150 daily median V’CO2 test days (tests), with an average of ~5 tests per patient. Each of these tests represented a calendar day with at least 60 once-per-minute V’CO2 measurements, from which the daily median was calculated. For these test days, the median number of V’CO2 measurements was 1431 (IQR 1217–1440) per day. The median age of patients was 66 (IQR 60–72) years, and patients were predominantly male (20, 65%, table 1). Overall, 10 (32%) patients were identified with an episode of >20% increase of median V’CO2 from 1 day to the next (table 1). Cardiovascular diseases, especially arterial hypertension, were more predominant in the group without a clinically relevant V’CO2 increase of >20%. Overall, differences in demographics and comorbidities of patients with or without V’CO2 increase were not observed beyond chance, with a similarity in comorbidities reflected by almost the same Charlson Comorbidity Score (table 1). To aid interpretation and assess the generalisability of our findings, we compared baseline characteristics of the included study population with those of the non-included ICU patients from the same source cohort.

**Table 1:** Baseline characteristics

	Overall (N=31)	No significant V’CO2 increase (n=21)	V’CO2 increase (n=10)	P value
Baseline characteristics				
Age (years), median (IQR)	66 (60–72)	68 (60–72)	65.5 (54–71)	0.7
Female sex, n (%)	11 (35.5%)	7 (33.3%)	4 (36.4%)	0.9
Pregnant females	0 (0%)	0 (0%)	0 (0%)	–
Body mass index (kg/m^2^)	26.8 (24.2–33.4)	26.9 (24.2–33.4)	25.7 (24.4–30.9)	0.6
Coronary heart disease/myocardial infarction	3 (9.7%)	3 (14.29%)	0 (0%)	0.5
Arterial hypertension	20 (64.5%)	15 (71.4%)	5 (50.0%)	0.1
Diabetes mellitus	9 (29.0%)	6 (28.57%)	3 (30.0%)	0.2
Chronic obstructive pulmonary disease	1 (3.2%)	0 (0%)	1 (10.0%)	0.2
Asthma	2 (6.5%)	1 (4.8%)	1 (10.0%)	0.7
Obstructive sleep apnoea	2 (6.5%)	2 (9.5%)	0 (0%)	0.3
Chronic kidney disease stage I-V KDIGO 2012	2 (6.5%)	1 (4.8%)	1 (10.0%)	0.7
Solid organ transplant	1 (3.2%)	1 (4.8%)	0 (0%)	0.5
Immunosuppression	2 (6.5%)	2 (9.5%)	0 (0%)	0.3
Cancer	4 (12.9%)	3 (14.3%)	1 (10.0%)	0.6
Scores at baseline				
Sequential Organ Failure Assessment score	7 (3–9)	7 (3–9)	8 (3–10)	0.6
Simplified Acute Physiology Score II	35 (27–40)	35 (30–37)	37.5 (25–44)	0.3
Charlson Comorbidity Score	1 (0–2)	1 (0–2)	0.5 (0–2)	0.6
COVID-19 targeted therapy				
Dexamethasone therapy	26 (83.9%)	17 (81.0%)	9 (90.0%)	0.8
Remdesivir therapy	13 (41.9%)	10 (47.6%)	3 (30.0%)	0.6
Plasma therapy	0 (0%)	0 (0%)	0 (0%)	–

Demographic and clinical characteristics as well as risk factors of COVID-19 patients stratified according to presence or absence of a first episode of V’CO2 increase of 20% or more within 1 day. The data are presented as median (IQR) or number (percentage).

KDIGO, kidney disease: improving global outcomes; V’CO2, exhaled carbon dioxide.

The 31 patients included in the study differed from the non-included patients in terms of proportion intubated (100% in included vs 70% in non-included) and dexamethasone treatment at baseline (84% in included vs 56% in non-included), while other baseline characteristics did not differ substantially (online supplemental table 1).

### Description of V’CO2 increase and superinfection

Overall, median daily V’CO2 was substantially higher in patients who eventually developed a superinfection during ICU stay, with 210 mL/min (IQR 175–247) compared with 176 mL/min (IQR 153–204) in those who did not (p<0.001), based on all eligible days prior to the first superinfection.

A first bacterial superinfection occurred in 12 patients (39%) after a median of 6.5 days of V’CO2 monitoring (IQR 3.5–8). Pulmonary superinfection was detected in 92% of cases, with 25% of patients developing bacteraemia, mostly in addition to pulmonary involvement. Only one patient (8%) had isolated bacteraemia.

**Figure 2:**
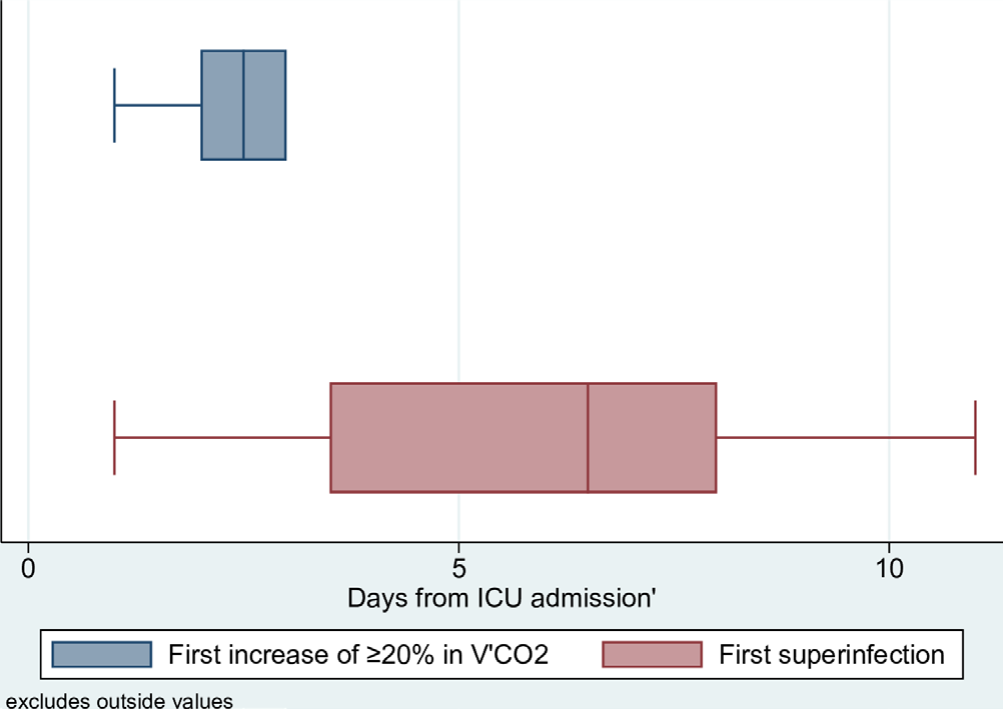
Time from ICU admission to first V’CO2 increase >20% and first superinfection, respectively. Time point of ICU admission to first detection of a >20% daily V’CO2 increase compared with the following day (blue) and time point of first bacterial superinfection (red). The bar plot represents the upper and lower quartiles with median, the whiskers represent variability outside the IQR. ICU, intensive care unit; V’CO2, exhaled carbon dioxide.

**Figure 3:**
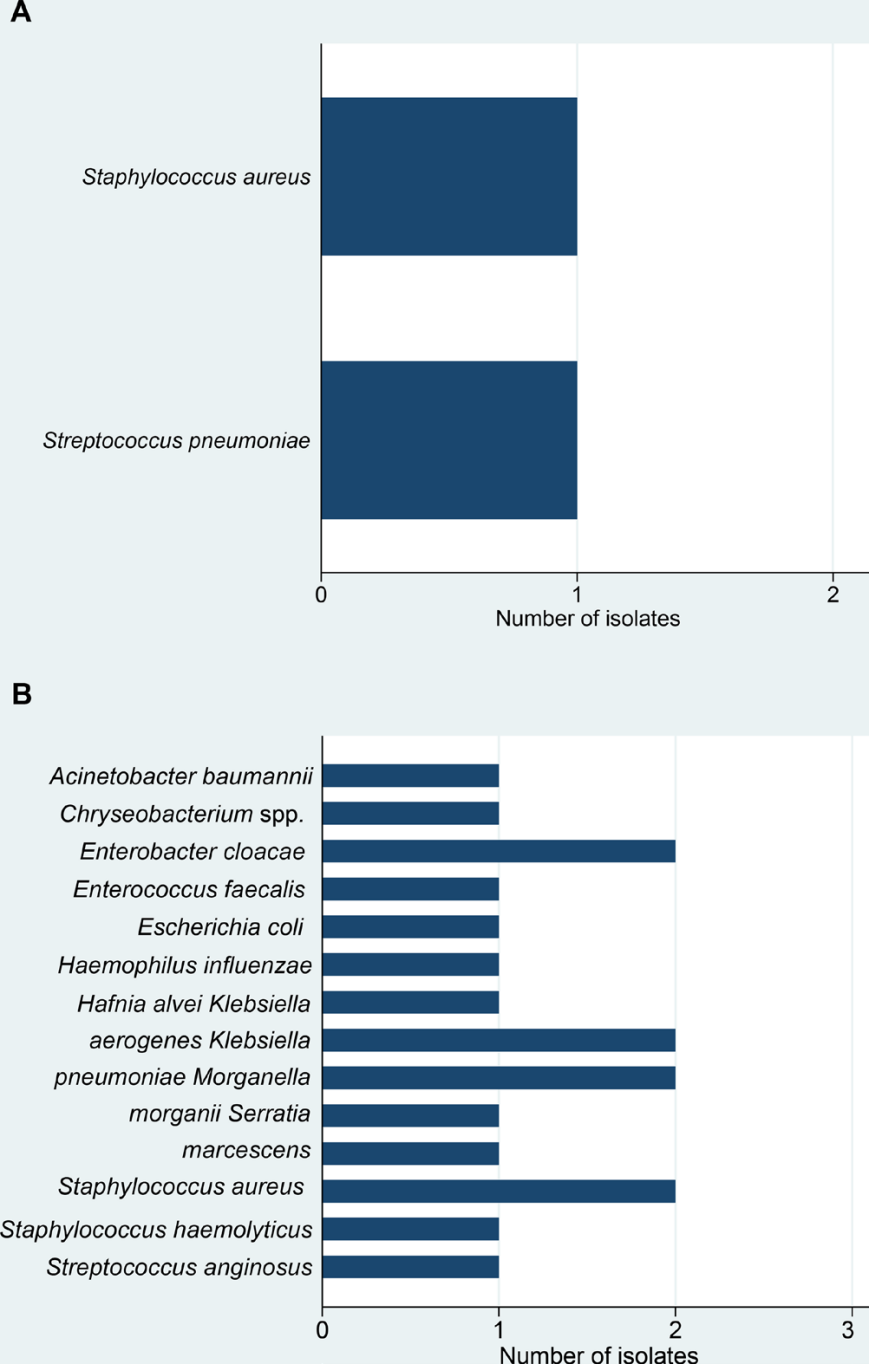
(**A**) Isolated bacteria in patients with a V’CO2 increase >20%. All bacteria detected in any sample in the group with a >20% V’CO2 increase from 1 day to the next. Data are presented in absolute numbers. (**B**) Isolated bacteria from patients without V’CO2 increase >20%. All bacteria detected in any sample in the group without a >20% V’CO2 increase from 1 day to the next. Data are presented in absolute numbers. V’CO2, exhaled carbon dioxide.

In a total of 10 (32%) patients, a first episode of a substantial V’CO2 increase of >20% was observed after a median of 2.5 (IQR 2–3) days (figure 2), while the occurrence of a superinfection on the day of a V’CO2 increase was only seen in two patients. In these patients with a V’CO2 increase on the day of superinfection, the isolated bacteria were *Staphylococcus aureus* and *Streptococcus pneumoniae* (figure 3A). The overall spectrum of isolated bacteria was broad, with the most common being *S. aureus*, *Klebsiella pneumoniae*, *Klebsiella aerogenes* and *Enterobacter cloacae* in patients without substantial V’CO2 increase on the day of superinfection (figure 3B).

### Association of V’CO2 and superinfection

A first V’CO2 increase of >20% and the occurrence of a first superinfection was not associated beyond chance (OR 3.47, 95% CI 0.64 to 18.92, p=0.15), independent of age and sex. This did not differ substantially from a crude analysis (OR 3.25, 95% CI 0.61 to 17.4, p=0.17), or when a superinfection on the same as well as the next day was considered in a sensitivity analysis.

However, when looking at the absolute levels of V’CO2 as opposed to only a sudden increase, daily median V’CO2 was strongly associated with developing a superinfection during ICU stay with an age and sex adjusted OR of 1.56 (95% CI 1.16 to 2.08, p=0.003) per 50 mL/min (daily average) increase in V’CO2.

### Sensitivity, specificity and use of V’CO2 increase to detect superinfections

During a total of 150 tests, defined as days with a median V’CO2 that were eligible for analysis where patients could have developed a superinfection, we observed 2 true positive tests and 10 false negative tests, while 130 were true negative and 8 false positive. Therefore, the sensitivity of a daily median V’CO2 increase of >20% to detect a bacterial superinfection was 17% (95% CI 2% to 48%), with a specificity of 94% (95% CI 89% to 98%). While the LR+ to detect a bacterial superinfection was 2.88 (95% CI 0.69 to 12.05), the LR– was only 0.88 (95% CI 0.68 to 1.14), resulting in an overall limited performance of a sudden increase in V’CO2 to detect superinfections.

## Discussion

In this prospective cohort study of invasively ventilated patients with ARDS due to SARS-CoV-2 and V’CO2 monitoring, we did not find clear evidence of an association between an immediate increase of >20% in daily median V’CO2 and a bacterial superinfection on that same day, but found a strong association of higher daily median V’CO2 levels and developing a superinfection during ICU stay.

The latter was reflected in a low sensitivity (17%) to detect a superinfection in the presence of a relevant V’CO2 increase at a >20% cut-off compared with the previous day. However, the specificity of 94% was fairly high, which resulted in an LR+ of 2.88, thus still increasing the odds for a bacterial superinfection when observing a first relevant V’CO2 increase by almost three times.

In our study, the profile of pathogens implicated in causing superinfection was similar to results reported in prior studies. The presence of *Staphylococcus aureus* and *Streptococcus pneumoniae* in patients who had an increase in V'CO2 on the same day as the onset of superinfection was not unexpected, given their virulence and potential for a fulminant course of disease.

Our study is—to our knowledge—the first to investigate the association of V’CO2 and a bacterial superinfection in mechanically ventilated patients with ARDS. The poor sensitivity of a 20% daily median V’CO2 increase despite an overall higher V’CO2 level in patients with superinfection may be due to an overall hypermetabolic state in these patients alone,^18 19^ and thus the additional carbon dioxide production from a gradually progressing superinfection might not increase sharply enough to lead to a sudden relevant increase in daily median V’CO2 from 1 day to the other.

On the other hand, it has long been recognised that V’CO2 is influenced by multiple factors. Those include, for example, changes in ventilation such as minute ventilation, breathing pattern, ventilation pressures, high inspiratory oxygen concentrations (above 60%), sedation status and the metabolic production of CO2 related to nutrition.^9^ In particular, to reach a steady state of V’CO2 after any change in ventilation, it usually takes from 20 to 120 min.^9 27^ Thus, despite only using median daily V’CO2 changes to reduce noise from short-term variations, we suspect these other (in our study unmeasured) factors could also have influenced the sensitivity for a 20% V’CO2 increase to detect a superinfection in our study. Furthermore, it could also be argued that the lack of a clear cut-off in V’CO2 increase despite overall higher V’CO2 values in patients who subsequently developed a superinfection may be in part because hourly within-patient variation in the advent of a superinfection may not translate to a sharp between-day variation. However, because the exact hour of superinfection onset is unknown, we were unable to investigate an association of hourly median V’CO2 changes and superinfection. Given the low sensitivity but high specificity, we consider continuous V’CO2 monitoring not as a stand-alone diagnostic method, but as a supportive tool that may aid early recognition when interpreted alongside clinical and microbiological findings.

Our study has several strengths. First, the prospective setting with longitudinal sampling of respiratory specimens and simultaneous continuous recording of ventilator data, demographic data and standardised microbiological evaluations. This is likely to have reduced detection bias, where patients otherwise might preferably be sampled only when clinical status deteriorates, a condition also potentially associated with an increase in V’CO2. Second, the study took place in a tertiary care centre in a high-resource setting without healthcare shortages during the first and second pandemic waves examined in this study. Third, the prospective definition of a superinfection was strict and events were adjudicated. Fourth, we only included patients of the first and second pandemic wave, where the need for mechanical ventilation was predominantly due to COVID-19 ARDS alone. Finally, our study investigated a new and potentially innovative approach to aid the detection of superinfections in ARDS patients.

Our study also has several limitations. First, the single-centre study design and the requirement of available V’CO2 measurements limited the number of patients and events, resulting in a rather small sample size and possibly insufficient power to detect an association between a sudden increase of daily median V’CO2 and onset of a superinfection. However, for the calculation of sensitivity and specificity, the cumulative number of tests performed still amounted to a total of 150 tests.

Second, it is unlikely that V’CO2 has been measured during steady-state conditions in most of the measurements, potentially leading to a lack of precision to detect a clear cut-off and making it prone to influence from unmeasured factors, including changes in ventilation mode, adaptations in paCO2 targets by the treating clinicians, or changes in lung function, just to name the most obvious ones. Third, we were not able to trace the onset of a superinfection to an exact hour, making it impossible to investigate a relationship between hourly medians of V’CO2 and the onset of a superinfection. We have tried to mitigate this, and the potentially non-steady state use of V’CO2 measurements, by aggregating them to daily median values, which is, however, unlikely to completely resolve the issue. Thus, we consider these factors to be possible reasons for the lack of a clear cut-off in V’CO2 to detect a superinfection in our study. Fourth, we did not measure nutritional status. V’CO2 increases with a better nutritional status, and nutrition may have a positive influence on patient outcomes, including superinfections in critically ill patients. Thus, nutrition could potentially be an important unmeasured confounder of the association between V’CO2 and bacterial superinfections, potentially lowering the strength of an association between V’CO2 and superinfections. Fifth, in order to investigate a somewhat homogenous population, we included only patients with ARDS due to SARS-CoV-2; it remains unclear to what extent our results can be generalised to patients with ARDS due to a different cause.

Finally, we regarded our study as exploratory in its nature and did not adjust for multiple hypothesis testing, and while we did find strong evidence for an association between higher daily median V’CO2 values and the subsequent development of a superinfection, this relationship may not be causal.

## Conclusions

In conclusion, we did not find a sudden clinically relevant increase in daily median V’CO2 of >20% to be associated with the onset of a bacterial superinfection on the same day, despite strong evidence for higher daily median V’CO2 levels in patients who developed a superinfection. The high specificity of a sudden V'CO2 increase in detecting superinfection suggests that a significant daily rise in V'CO2 should prompt consideration of a bacterial superinfection. However, given the poor sensitivity observed, we do not propose daily V’CO2 increases as a stand-alone diagnostic method for pulmonary superinfections. Rather, we suggest it may serve as a supportive tool to raise clinical suspicion when integrated with other clinical, microbiological and radiological findings. Our results may help inform future management of ARDS and highlight the need for further research, ideally using a larger sample size and more diverse patient populations.

## Supplementary material

10.1136/bmjresp-2024-002760 online supplemental file 1

## Data Availability

Data are available on reasonable request.
