# Supplementary material for: Use of carbon dioxide production to detect bacterial superinfections in mechanically ventilated patients with acute respiratory distress syndrome: an exploratory prospective cohort study
Source: BMJ Open Respir Res. 2025 Aug 28;12(1):e002760. doi: 10.1136/bmjresp-2024-002760 (PMC12410624; doi:10.1136/bmjresp-2024-002760)
Supplement: online supplemental file 1 [file bmjresp-12-1-s001.docx]

## Supplementary Table 1: Baseline characteristics for patients included and not included.

|  | **Overall**  **(N=189)** | **Included**  **(n=31)** | **Not included^1^ (n=158)** | **p-value** |
| --- | --- | --- | --- | --- |
| **Baseline characteristics** | |  | | |
| Age (years), median (IQR) | 64 (54-72) | 66 (60-72) | 64 (54-72) | 0.4 |
| Female sex, n (%) | 50 (26%) | 11 (36%) | 39 (25%) | 0.2 |
| Pregnant females | 2 (1%) | 0 (0%) | 2 (3%) | >0.9 |
| Body mass index (kg/m2) | 28 (25-32) | 26.8 (24.2-33.4) | 28 (25-32) | 0.9 |
| Coronary heart disease | 41 (22%) | 3 (10%) | 38 (25%) | 0.095 |
| Arterial hypertension | 108 (59%) | 20 (65%) | 88 (58%) | 0.5 |
| Diabetes mellitus | 68 (37%) | 9 (29%) | 59 (39%) | 0.3 |
| Chronic obstructive pulmonary disease | 17 (9%) | 1 (3%) | 16 (10) | 0.3 |
| Asthma | 12 (7%) | 2 (6%) | 10 (7) | >0.9 |
| Obstructive sleep apnea | 11 (12%) | 2 (6%) | 8 (13) | 0.7 |
| Chronic kidney disease stage I-V KDIGO 2012 | 33 (18%) | 2 (6%) | 31 (20) | 0.08 |
| Solid organ transplant | 10 (5%) | 1 (3%) | 9 (6%) | >0.9 |
| Immunosuppression | 17 (9%) | 2 (6%) | 15 (10%) | 0.7 |
| Cancer | 22 (12%) | 4 (13%) | 18 (12%) | 0.8 |
| Intubated at ICU admission | 141 (75%) | 31 (100%) | 110 (70%) | **<0.001*** |
| **Scores at baseline** | |  | | |
| Sequential organ failure assessment score (SOFA) | 7 (4-9) | 7 (3-9) | 6 (4-9) | 0.7 |
| Simplified Acute Physiology Score (SAPS) II | 37 (27-47) | 35 (27-40) | 38 (27-50) | 0.2 |
| Charlson Comorbidity Score | 1 (0-2) | 1 (0-2) | 1 (0-3) | 0.4 |
| **COVID-19 targeted therapy** | |  | | |
| Dexamethasone therapy | 75 (64 %) | 26 (84%) | 49 (56%) | **0.006*** |
| Remdesivir therapy | 45 (35%) | 13 (42%) | 32 (33%) | 0.4 |
| Plasma therapy | 2 (2%) | 0 (0%) | 2 (3%) | >0.9 |

Demographic and clinical characteristics as well as risk factors of COVID-19 patients stratified according to inclusion in the final V’CO2 analysis. The data are presented as median (interquartile range, IQR) or number (percentage).

* P-value “statistically significant”

1. Patients screened (n=147) or excluded (n=11).
